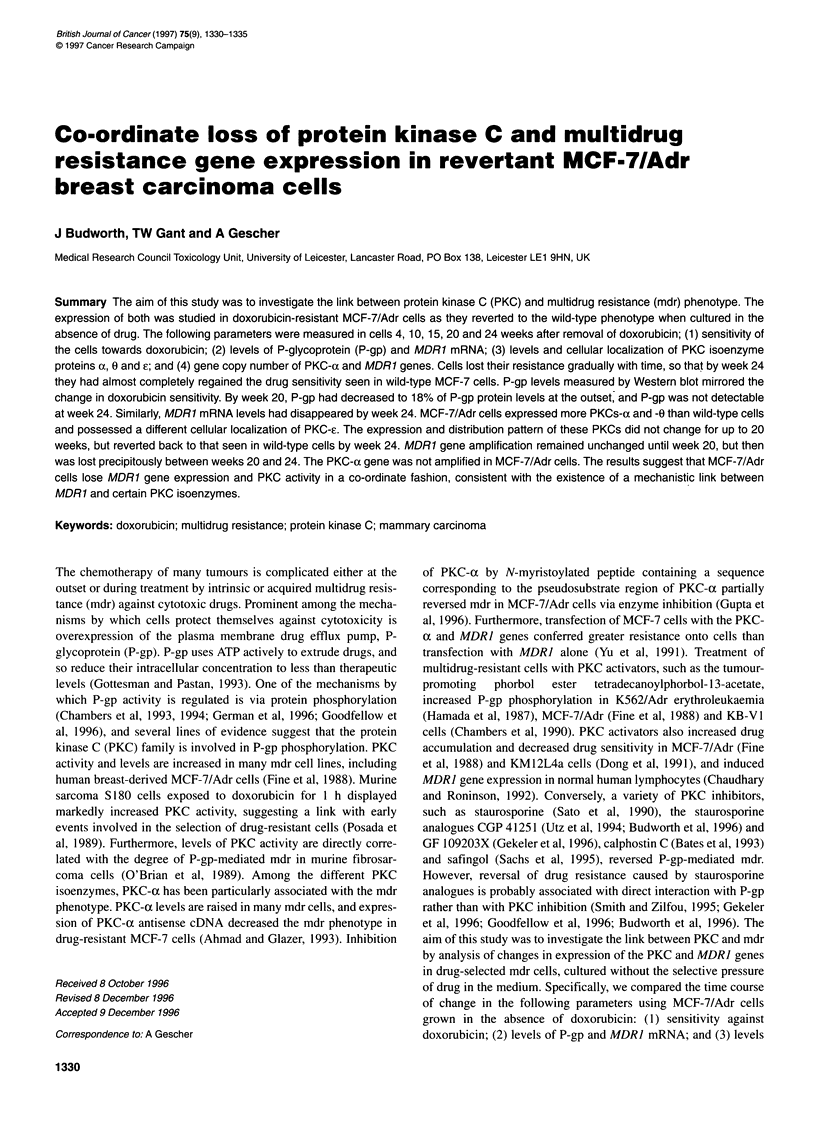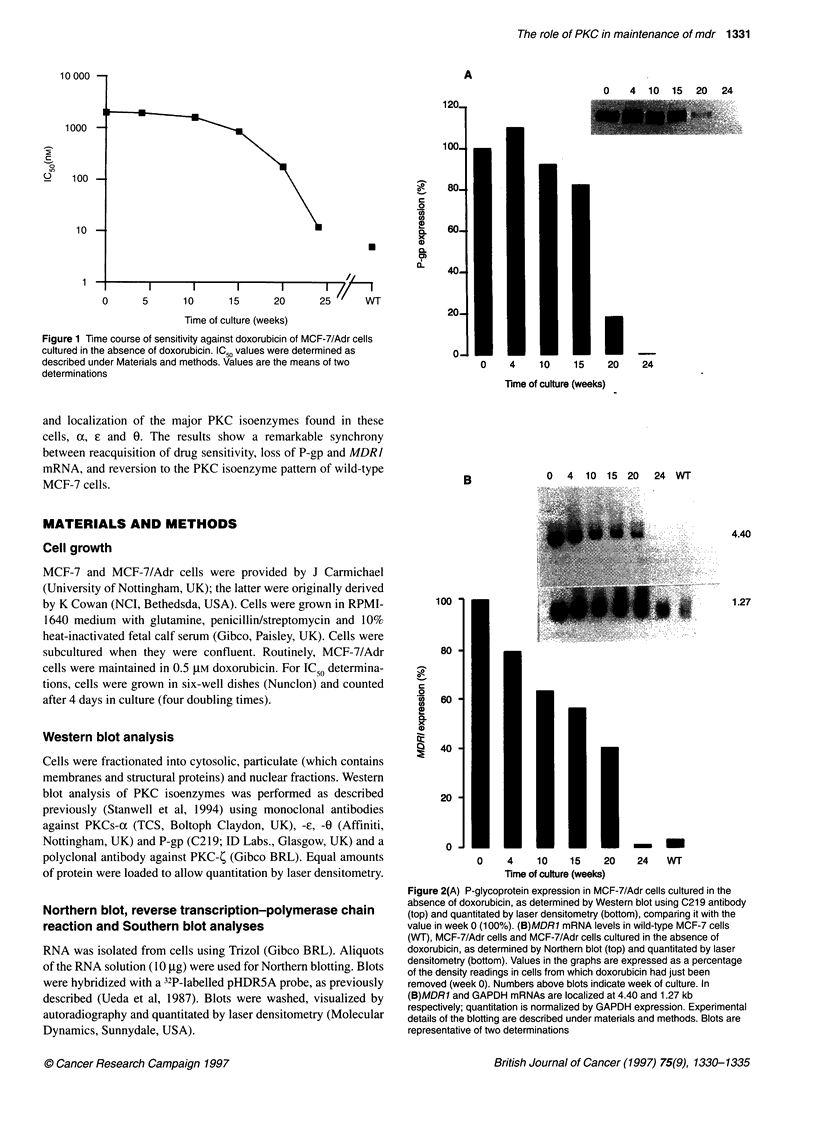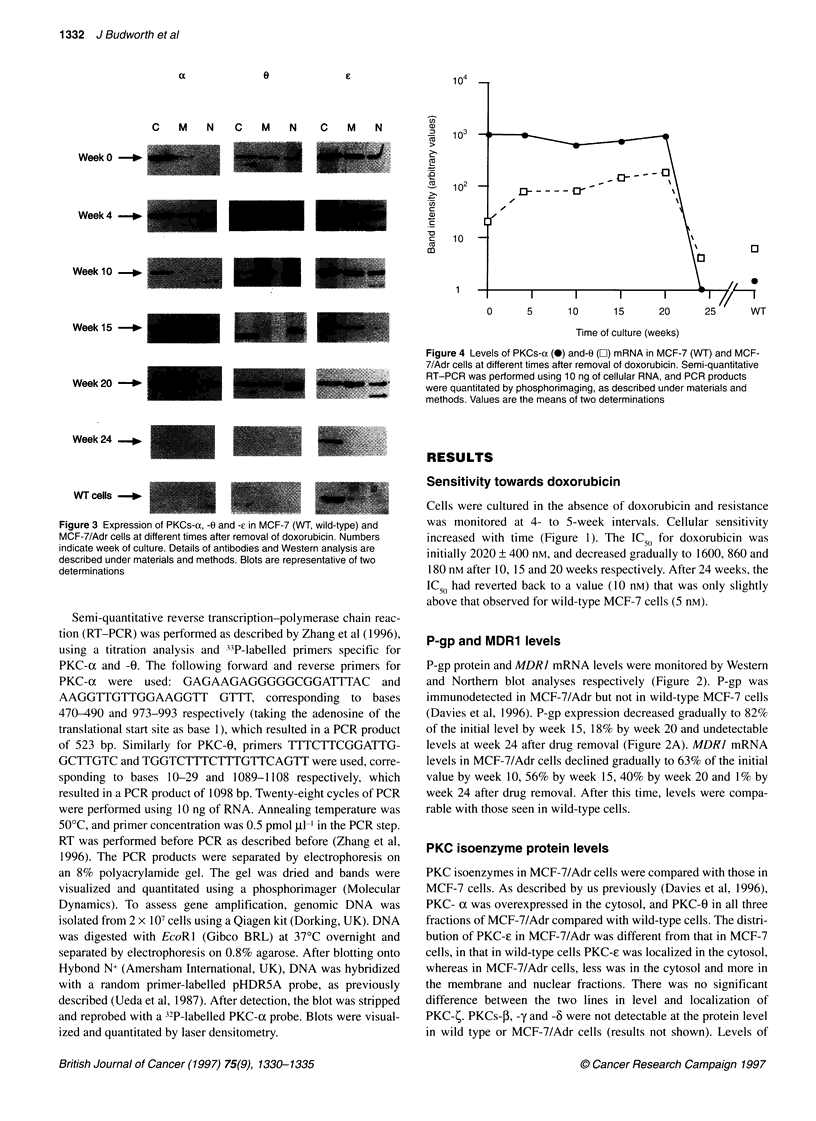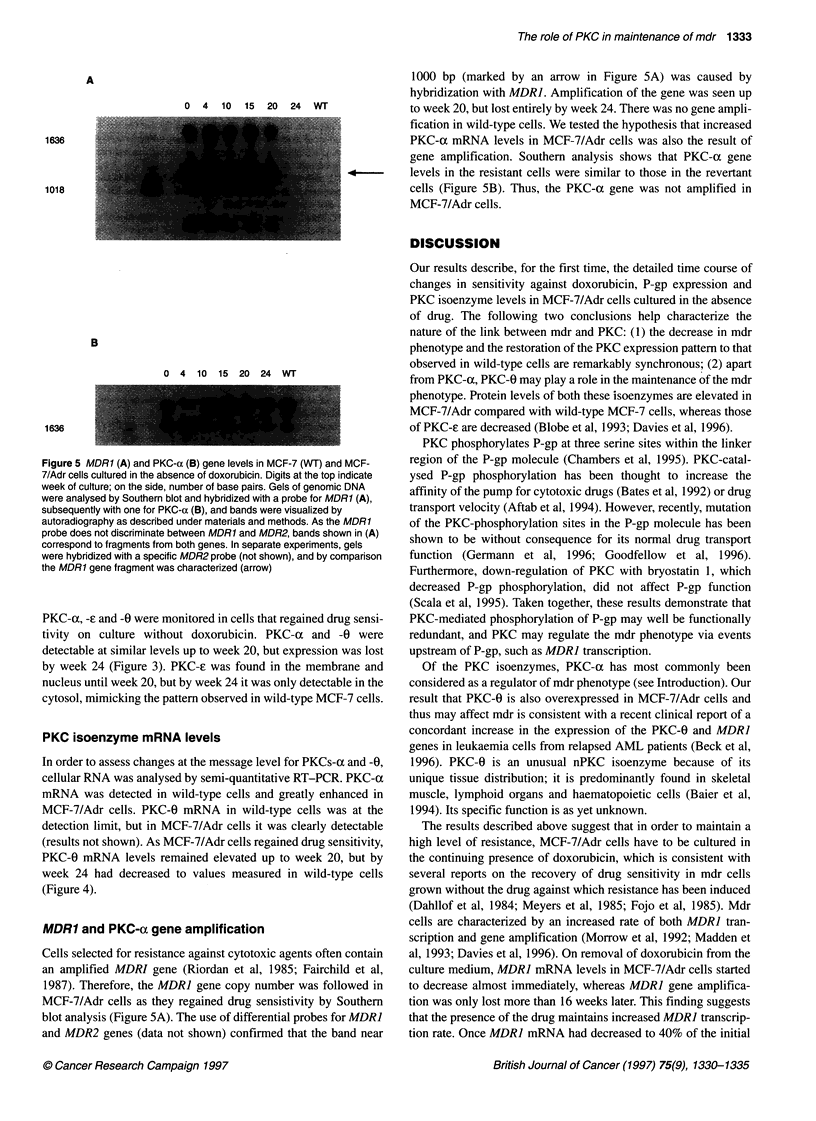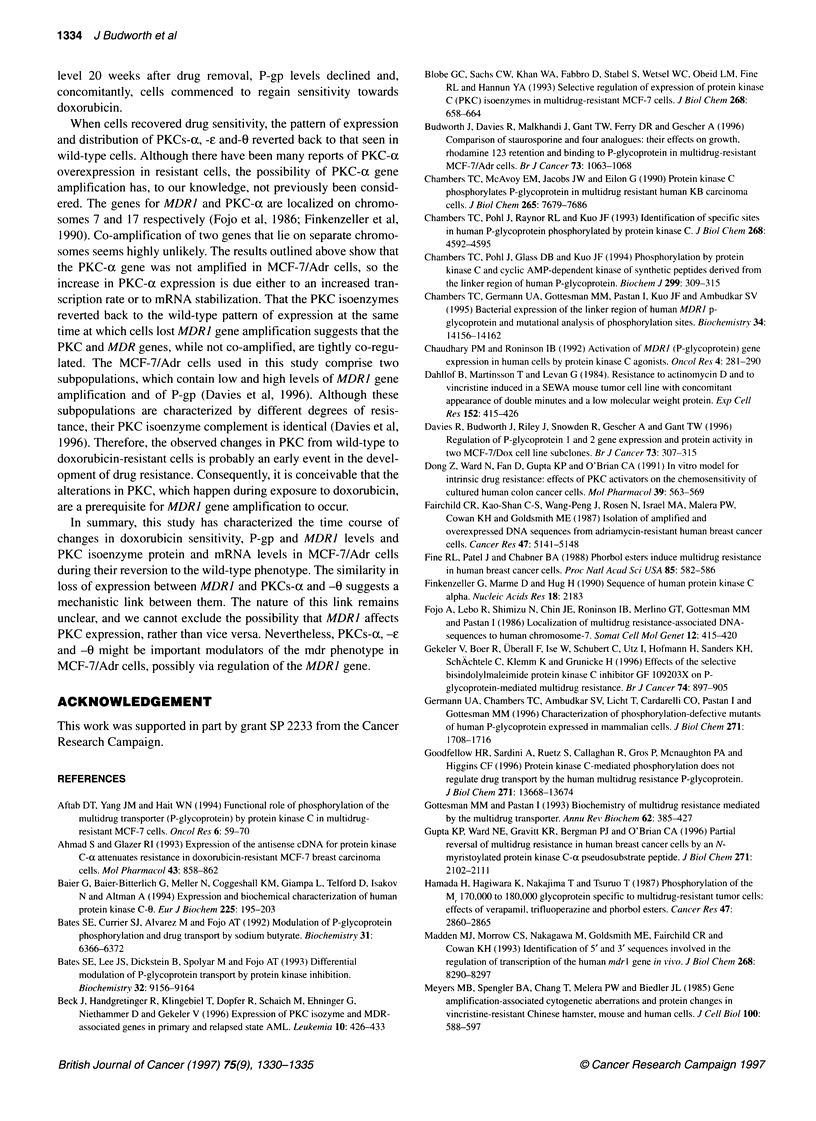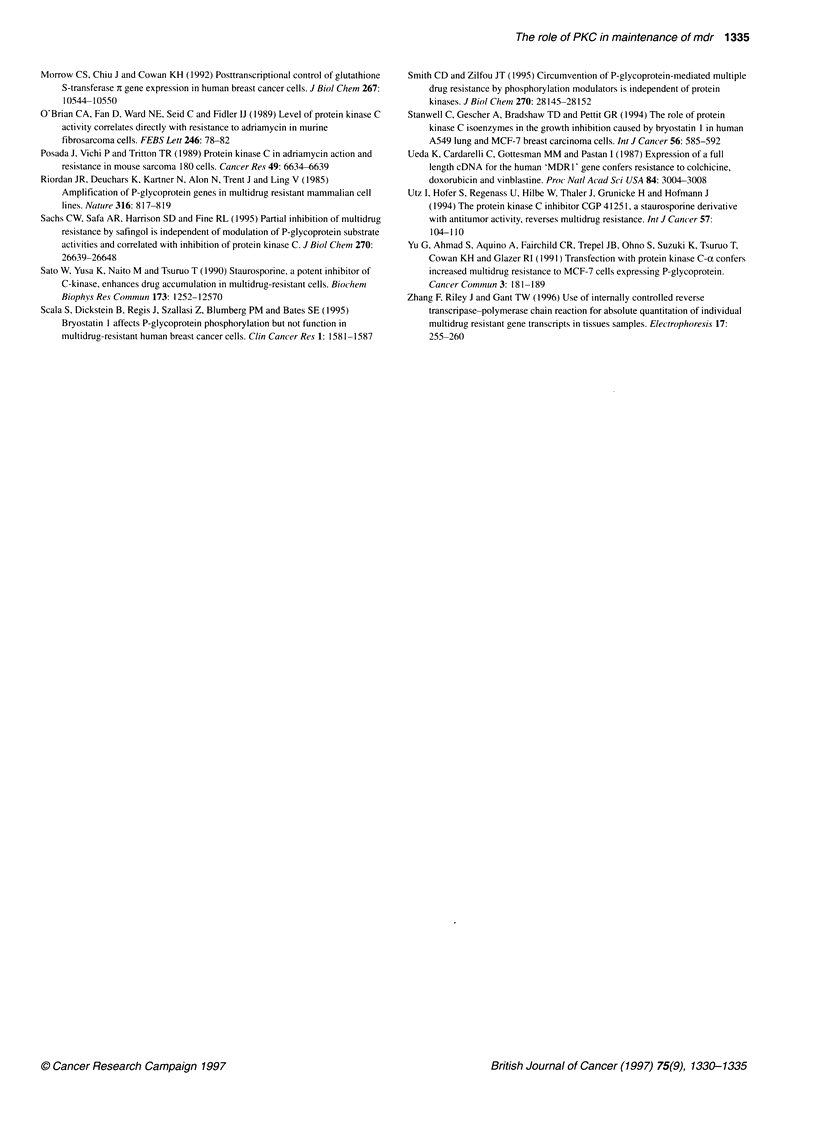# Co-ordinate loss of protein kinase C and multidrug resistance gene expression in revertant MCF-7/Adr breast carcinoma cells.

**DOI:** 10.1038/bjc.1997.225

**Published:** 1997

**Authors:** J. Budworth, T. W. Gant, A. Gescher

**Affiliations:** Medical Research Council Toxicology Unit, University of Leicester, UK.

## Abstract

**Images:**


British Journal of Cancer (1997) 75(9), 1330–1335
© 1997 Cancer Research Campaign

### Co-ordinate loss of protein kinase C and multidrug resistance gene expression in revertant MCF-7/Adr breast carcinoma cells

**J Budworth, TW Gant and A Gescher**

Medical Research Council Toxicology Unit, University of Leicester, Lancaster Road, PO Box 138, Leicester LE1 9HN, UK

**Summary** The aim of this study was to investigate the link between protein kinase C (PKC) and multidrug resistance (mdr) phenotype. The expression of both was studied in doxorubicin-resistant MCF-7/Adr cells as they reverted to the wild-type phenotype when cultured in the absence of drug. The following parameters were measured in cells 4, 10, 15, 20 and 24 weeks after removal of doxorubicin; (1) sensitivity of the cells towards doxorubicin; (2) levels of P-glycoprotein (P-gp) and *MDR1* mRNA; (3) levels and cellular localization of PKC isoenzyme proteins α, θ and ε; and (4) gene copy number of PKC-α and *MDR1* genes. Cells lost their resistance gradually with time, so that by week 24 they had almost completely regained the drug sensitivity seen in wild-type MCF-7 cells. P-gp levels measured by Western blot mirrored the change in doxorubicin sensitivity. By week 20, P-gp had decreased to 18% of P-gp protein levels at the outset, and P-gp was not detectable at week 24. Similarly, *MDR1* mRNA levels had disappeared by week 24. MCF-7/Adr cells expressed more PKCs-α and -θ than wild-type cells and possessed a different cellular localization of PKC-ε. The expression and distribution pattern of these PKCs did not change for up to 20 weeks, but reverted back to that seen in wild-type cells by week 24. *MDR1* gene amplification remained unchanged until week 20, but then was lost precipitously between weeks 20 and 24. The PKC-α gene was not amplified in MCF-7/Adr cells. The results suggest that MCF-7/Adr cells lose *MDR1* gene expression and PKC activity in a co-ordinate fashion, consistent with the existence of a mechanistic link between *MDR1* and certain PKC isoenzymes.

**Keywords:** doxorubicin; multidrug resistance; protein kinase C; mammary carcinoma

The chemotherapy of many tumours is complicated either at the outset or during treatment by intrinsic or acquired multidrug resistance (mdr) against cytotoxic drugs. Prominent among the mechanisms by which cells protect themselves against cytotoxicity is overexpression of the plasma membrane drug efflux pump, P-glycoprotein (P-gp). P-gp uses ATP actively to extrude drugs, and so reduce their intracellular concentration to less than therapeutic levels (Gottesman and Pastan, 1993). One of the mechanisms by which P-gp activity is regulated is via protein phosphorylation (Chambers et al, 1993, 1994; German et al, 1996; Goodfellow et al, 1996), and several lines of evidence suggest that the protein kinase C (PKC) family is involved in P-gp phosphorylation. PKC activity and levels are increased in many mdr cell lines, including human breast-derived MCF-7/Adr cells (Fine et al, 1988). Murine sarcoma S180 cells exposed to doxorubicin for 1 h displayed markedly increased PKC activity, suggesting a link with early events involved in the selection of drug-resistant cells (Posada et al, 1989). Furthermore, levels of PKC activity are directly correlated with the degree of P-gp-mediated mdr in murine fibrosarcoma cells (O'Brian et al, 1989). Among the different PKC isoenzymes, PKC-α has been particularly associated with the mdr phenotype. PKC-α levels are raised in many mdr cells, and expression of PKC-α antisense cDNA decreased the mdr phenotype in drug-resistant MCF-7 cells (Ahmad and Glazer, 1993). Inhibition of PKC-α by *N*-myristoylated peptide containing a sequence corresponding to the pseudosubstrate region of PKC-α partially reversed mdr in MCF-7/Adr cells via enzyme inhibition (Gupta et al, 1996). Furthermore, transfection of MCF-7 cells with the PKC-α and *MDR1* genes conferred greater resistance onto cells than transfection with *MDR1* alone (Yu et al, 1991). Treatment of multidrug-resistant cells with PKC activators, such as the tumour-promoting phorbol ester tetradecanoylphorbol-13-acetate, increased P-gp phosphorylation in K562/Adr erythroleukaemia (Hamada et al, 1987), MCF-7/Adr (Fine et al, 1988) and KB-V1 cells (Chambers et al, 1990). PKC activators also increased drug accumulation and decreased drug sensitivity in MCF-7/Adr (Fine et al, 1988) and KM12L4a cells (Dong et al, 1991), and induced *MDR1* gene expression in normal human lymphocytes (Chaudhary and Roninson, 1992). Conversely, a variety of PKC inhibitors, such as staurosporine (Sato et al, 1990), the staurosporine analogues CGP 41251 (Utz et al, 1994; Budworth et al, 1996) and GF 109203X (Gekeler et al, 1996), calphostin C (Bates et al, 1993) and safingol (Sachs et al, 1995), reversed P-gp-mediated mdr. However, reversal of drug resistance caused by staurosporine analogues is probably associated with direct interaction with P-gp rather than with PKC inhibition (Smith and Zilfou, 1995; Gekeler et al, 1996; Goodfellow et al, 1996; Budworth et al, 1996). The aim of this study was to investigate the link between PKC and mdr by analysis of changes in expression of the PKC and *MDR1* genes in drug-selected mdr cells, cultured without the selective pressure of drug in the medium. Specifically, we compared the time course of change in the following parameters using MCF-7/Adr cells grown in the absence of doxorubicin: (1) sensitivity against doxorubicin; (2) levels of P-gp and *MDR1* mRNA; and (3) levels

*Received 8 October 1996*
*Revised 8 December 1996*
*Accepted 9 December 1996*

*Correspondence to:* A Gescher

**Figure 1** Time course of sensitivity against doxorubicin of MCF-7/Adr cells cultured in the absence of doxorubicin. $IC_{50}$ values were determined as described under Materials and methods. Values are the means of two determinations and localization of the major PKC isoenzymes found in these cells, α, ε and θ. The results show a remarkable synchrony between reacquisition of drug sensitivity, loss of P-gp and *MDR1* mRNA, and reversion to the PKC isoenzyme pattern of wild-type MCF-7 cells.

### MATERIALS AND METHODS

### Cell growth

MCF-7 and MCF-7/Adr cells were provided by J Carmichael (University of Nottingham, UK); the latter were originally derived by K Cowan (NCI, Bethedsda, USA). Cells were grown in RPMI-1640 medium with glutamine, penicillin/streptomycin and 10% heat-inactivated fetal calf serum (Gibco, Paisley, UK). Cells were subcultured when they were confluent. Routinely, MCF-7/Adr cells were maintained in 0.5 μM doxorubicin. For $IC_{50}$ determinations, cells were grown in six-well dishes (Nunclon) and counted after 4 days in culture (four doubling times).

### Western blot analysis

Cells were fractionated into cytosolic, particulate (which contains membranes and structural proteins) and nuclear fractions. Western blot analysis of PKC isoenzymes was performed as described previously (Stanwell et al, 1994) using monoclonal antibodies against PKCs-α (TCS, Boltoph Claydon, UK), -ε, -θ (Affiniti, Nottingham, UK) and P-gp (C219; ID Labs., Glasgow, UK) and a polyclonal antibody against PKC-ζ (Gibco BRL). Equal amounts of protein were loaded to allow quantitation by laser densitometry.

### Northern blot, reverse transcription–polymerase chain reaction and Southern blot analyses

RNA was isolated from cells using Trizol (Gibco BRL). Aliquots of the RNA solution (10 μg) were used for Northern blotting. Blots were hybridized with a $^{32}$P-labelled pHDR5A probe, as previously described (Ueda et al, 1987). Blots were washed, visualized by autoradiography and quantitated by laser densitometry (Molecular Dynamics, Sunnydale, USA).

**Figure 2**(A) P-glycoprotein expression in MCF-7/Adr cells cultured in the absence of doxorubicin, as determined by Western blot using C219 antibody (top) and quantitated by laser densitometry (bottom), comparing it with the value in week 0 (100%). (**B**)*MDR1* mRNA levels in wild-type MCF-7 cells (WT), MCF-7/Adr cells and MCF-7/Adr cells cultured in the absence of doxorubicin, as determined by Northern blot (top) and quantitated by laser densitometry (bottom). Values in the graphs are expressed as a percentage of the density readings in cells from which doxorubicin had just been removed (week 0). Numbers above blots indicate week of culture. In (**B**)*MDR1* and GAPDH mRNAs are localized at 4.40 and 1.27 kb respectively; quantitation is normalized by GAPDH expression. Experimental details of the blotting are described under materials and methods. Blots are representative of two determinations

© Cancer Research Campaign 1997

**Figure 3** Expression of PKCs-α, -θ and -ε in MCF-7 (WT, wild-type) and MCF-7/Adr cells at different times after removal of doxorubicin. Numbers indicate week of culture. Details of antibodies and Western analysis are described under materials and methods. Blots are representative of two determinations

**Figure 4** Levels of PKCs-α (●) and-θ (□) mRNA in MCF-7 (WT) and MCF-7/Adr cells at different times after removal of doxorubicin. Semi-quantitative RT–PCR was performed using 10 ng of cellular RNA, and PCR products were quantitated by phosphorimaging, as described under materials and methods. Values are the means of two determinations

Semi-quantitative reverse transcription–polymerase chain reaction (RT–PCR) was performed as described by Zhang et al (1996), using a titration analysis and $^{33}$P-labelled primers specific for PKC-α and -θ. The following forward and reverse primers for PKC-α were used: GAGAAGAGGGGGCGGATTTAC and AAGGTTGTTGGAAGGTT GTTT, corresponding to bases 470–490 and 973–993 respectively (taking the adenosine of the translational start site as base 1), which resulted in a PCR product of 523 bp. Similarly for PKC-θ, primers TTTCTTCGGATTG-GCTTGTC and TGGTCTTTCTTTGTTCAGTT were used, corresponding to bases 10–29 and 1089–1108 respectively, which resulted in a PCR product of 1098 bp. Twenty-eight cycles of PCR were performed using 10 ng of RNA. Annealing temperature was 50°C, and primer concentration was 0.5 pmol $\mu l^{-1}$ in the PCR step. RT was performed before PCR as described before (Zhang et al, 1996). The PCR products were separated by electrophoresis on an 8% polyacrylamide gel. The gel was dried and bands were visualized and quantitated using a phosphorimager (Molecular Dynamics). To assess gene amplification, genomic DNA was isolated from $2 \times 10^7$ cells using a Qiagen kit (Dorking, UK). DNA was digested with *Eco*R1 (Gibco BRL) at 37°C overnight and separated by electrophoresis on 0.8% agarose. After blotting onto Hybond N$^+$ (Amersham International, UK), DNA was hybridized with a random primer-labelled pHDR5A probe, as previously described (Ueda et al, 1987). After detection, the blot was stripped and reprobed with a $^{32}$P-labelled PKC-α probe. Blots were visualized and quantitated by laser densitometry.

### RESULTS

### Sensitivity towards doxorubicin

Cells were cultured in the absence of doxorubicin and resistance was monitored at 4- to 5-week intervals. Cellular sensitivity increased with time (Figure 1). The $IC_{50}$ for doxorubicin was initially 2020 ± 400 nM, and decreased gradually to 1600, 860 and 180 nM after 10, 15 and 20 weeks respectively. After 24 weeks, the $IC_{50}$ had reverted back to a value (10 nM) that was only slightly above that observed for wild-type MCF-7 cells (5 nM).

### P-gp and MDR1 levels

P-gp protein and *MDR1* mRNA levels were monitored by Western and Northern blot analyses respectively (Figure 2). P-gp was immunodetected in MCF-7/Adr but not in wild-type MCF-7 cells (Davies et al, 1996). P-gp expression decreased gradually to 82% of the initial level by week 15, 18% by week 20 and undetectable levels at week 24 after drug removal (Figure 2A). *MDR1* mRNA levels in MCF-7/Adr cells declined gradually to 63% of the initial value by week 10, 56% by week 15, 40% by week 20 and 1% by week 24 after drug removal. After this time, levels were comparable with those seen in wild-type cells.

### PKC isoenzyme protein levels

PKC isoenzymes in MCF-7/Adr cells were compared with those in MCF-7 cells. As described by us previously (Davies et al, 1996), PKC- α was overexpressed in the cytosol, and PKC-θ in all three fractions of MCF-7/Adr compared with wild-type cells. The distribution of PKC-ε in MCF-7/Adr was different from that in MCF-7 cells, in that in wild-type cells PKC-ε was localized in the cytosol, whereas in MCF-7/Adr cells, less was in the cytosol and more in the membrane and nuclear fractions. There was no significant difference between the two lines in level and localization of PKC-ζ. PKCs-β, -γ and -δ were not detectable at the protein level in wild type or MCF-7/Adr cells (results not shown). Levels of

© Cancer Research Campaign 1997

**Figure 5** *MDR1* (**A**) and PKC-α (**B**) gene levels in MCF-7 (WT) and MCF-7/Adr cells cultured in the absence of doxorubicin. Digits at the top indicate week of culture; on the side, number of base pairs. Gels of genomic DNA were analysed by Southern blot and hybridized with a probe for *MDR1* (**A**), subsequently with one for PKC-α (**B**), and bands were visualized by autoradiography as described under materials and methods. As the *MDR1* probe does not discriminate between *MDR1* and *MDR2*, bands shown in (**A**) correspond to fragments from both genes. In separate experiments, gels were hybridized with a specific *MDR2* probe (not shown), and by comparison the *MDR1* gene fragment was characterized (arrow)

PKC-α, -ε and -θ were monitored in cells that regained drug sensitivity on culture without doxorubicin. PKC-α and -θ were detectable at similar levels up to week 20, but expression was lost by week 24 (Figure 3). PKC-ε was found in the membrane and nucleus until week 20, but by week 24 it was only detectable in the cytosol, mimicking the pattern observed in wild-type MCF-7 cells.

### PKC isoenzyme mRNA levels

In order to assess changes at the message level for PKCs-α and -θ, cellular RNA was analysed by semi-quantitative RT–PCR. PKC-α mRNA was detected in wild-type cells and greatly enhanced in MCF-7/Adr cells. PKC-θ mRNA in wild-type cells was at the detection limit, but in MCF-7/Adr cells it was clearly detectable (results not shown). As MCF-7/Adr cells regained drug sensitivity, PKC-θ mRNA levels remained elevated up to week 20, but by week 24 had decreased to values measured in wild-type cells (Figure 4).

### *MDR1* and PKC-α gene amplification

Cells selected for resistance against cytotoxic agents often contain an amplified *MDR1* gene (Riordan et al, 1985; Fairchild et al, 1987). Therefore, the *MDR1* gene copy number was followed in MCF-7/Adr cells as they regained drug sensistivity by Southern blot analysis (Figure 5A). The use of differential probes for *MDR1* and *MDR2* genes (data not shown) confirmed that the band near 1000 bp (marked by an arrow in Figure 5A) was caused by hybridization with *MDR1*. Amplification of the gene was seen up to week 20, but lost entirely by week 24. There was no gene amplification in wild-type cells. We tested the hypothesis that increased PKC-α mRNA levels in MCF-7/Adr cells was also the result of gene amplification. Southern analysis shows that PKC-α gene levels in the resistant cells were similar to those in the revertant cells (Figure 5B). Thus, the PKC-α gene was not amplified in MCF-7/Adr cells.

### DISCUSSION

Our results describe, for the first time, the detailed time course of changes in sensitivity against doxorubicin, P-gp expression and PKC isoenzyme levels in MCF-7/Adr cells cultured in the absence of drug. The following two conclusions help characterize the nature of the link between mdr and PKC: (1) the decrease in mdr phenotype and the restoration of the PKC expression pattern to that observed in wild-type cells are remarkably synchronous; (2) apart from PKC-α, PKC-θ may play a role in the maintenance of the mdr phenotype. Protein levels of both these isoenzymes are elevated in MCF-7/Adr compared with wild-type MCF-7 cells, whereas those of PKC-ε are decreased (Blobe et al, 1993; Davies et al, 1996).

PKC phosphorylates P-gp at three serine sites within the linker region of the P-gp molecule (Chambers et al, 1995). PKC-catalysed P-gp phosphorylation has been thought to increase the affinity of the pump for cytotoxic drugs (Bates et al, 1992) or drug transport velocity (Aftab et al, 1994). However, recently, mutation of the PKC-phosphorylation sites in the P-gp molecule has been shown to be without consequence for its normal drug transport function (Germann et al, 1996; Goodfellow et al, 1996). Furthermore, down-regulation of PKC with bryostatin 1, which decreased P-gp phosphorylation, did not affect P-gp function (Scala et al, 1995). Taken together, these results demonstrate that PKC-mediated phosphorylation of P-gp may well be functionally redundant, and PKC may regulate the mdr phenotype via events upstream of P-gp, such as *MDR1* transcription.

Of the PKC isoenzymes, PKC-α has most commonly been considered as a regulator of mdr phenotype (see Introduction). Our result that PKC-θ is also overexpressed in MCF-7/Adr cells and thus may affect mdr is consistent with a recent clinical report of a concordant increase in the expression of the PKC-θ and *MDR1* genes in leukaemia cells from relapsed AML patients (Beck et al, 1996). PKC-θ is an unusual nPKC isoenzyme because of its unique tissue distribution; it is predominantly found in skeletal muscle, lymphoid organs and haematopoietic cells (Baier et al, 1994). Its specific function is as yet unknown.

The results described above suggest that in order to maintain a high level of resistance, MCF-7/Adr cells have to be cultured in the continuing presence of doxorubicin, which is consistent with several reports on the recovery of drug sensitivity in mdr cells grown without the drug against which resistance has been induced (Dahllof et al, 1984; Meyers et al, 1985; Fojo et al, 1985). Mdr cells are characterized by an increased rate of both *MDR1* transcription and gene amplification (Morrow et al, 1992; Madden et al, 1993; Davies et al, 1996). On removal of doxorubicin from the culture medium, *MDR1* mRNA levels in MCF-7/Adr cells started to decrease almost immediately, whereas *MDR1* gene amplification was only lost more than 16 weeks later. This finding suggests that the presence of the drug maintains increased *MDR1* transcription rate. Once *MDR1* mRNA had decreased to 40% of the initial

© Cancer Research Campaign 1997

level 20 weeks after drug removal, P-gp levels declined and, concomitantly, cells commenced to regain sensitivity towards doxorubicin.

When cells recovered drug sensitivity, the pattern of expression and distribution of PKCs-α, -ε and-θ reverted back to that seen in wild-type cells. Although there have been many reports of PKC-α overexpression in resistant cells, the possibility of PKC-α gene amplification has, to our knowledge, not previously been considered. The genes for *MDR1* and PKC-α are localized on chromosomes 7 and 17 respectively (Fojo et al, 1986; Finkenzeller et al, 1990). Co-amplification of two genes that lie on separate chromosomes seems highly unlikely. The results outlined above show that the PKC-α gene was not amplified in MCF-7/Adr cells, so the increase in PKC-α expression is due either to an increased transcription rate or to mRNA stabilization. That the PKC isoenzymes reverted back to the wild-type pattern of expression at the same time at which cells lost *MDR1* gene amplification suggests that the PKC and *MDR* genes, while not co-amplified, are tightly co-regulated. The MCF-7/Adr cells used in this study comprise two subpopulations, which contain low and high levels of *MDR1* gene amplification and of P-gp (Davies et al, 1996). Although these subpopulations are characterized by different degrees of resistance, their PKC isoenzyme complement is identical (Davies et al, 1996). Therefore, the observed changes in PKC from wild-type to doxorubicin-resistant cells is probably an early event in the development of drug resistance. Consequently, it is conceivable that the alterations in PKC, which happen during exposure to doxorubicin, are a prerequisite for *MDR1* gene amplification to occur.

In summary, this study has characterized the time course of changes in doxorubicin sensitivity, P-gp and *MDR1* levels and PKC isoenzyme protein and mRNA levels in MCF-7/Adr cells during their reversion to the wild-type phenotype. The similarity in loss of expression between *MDR1* and PKCs-α and –θ suggests a mechanistic link between them. The nature of this link remains unclear, and we cannot exclude the possibility that *MDR1* affects PKC expression, rather than vice versa. Nevertheless, PKCs-α, –ε and –θ might be important modulators of the mdr phenotype in MCF-7/Adr cells, possibly via regulation of the *MDR1* gene.

### ACKNOWLEDGEMENT

This work was supported in part by grant SP 2233 from the Cancer Research Campaign.

### REFERENCES

Aftab DT, Yang JM and Hait WN (1994) Functional role of phosphorylation of the multidrug transporter (P-glycoprotein) by protein kinase C in multidrug-resistant MCF-7 cells. *Oncol Res* **6**: 59–70

Ahmad S and Glazer RI (1993) Expression of the antisense cDNA for protein kinase C-α attenuates resistance in doxorubicin-resistant MCF-7 breast carcinoma cells. *Mol Pharmacol* **43**: 858–862

Baier G, Baier-Bitterlich G, Meller N, Coggeshall KM, Giampa L, Telford D, Isakov N and Altman A (1994) Expression and biochemical characterization of human protein kinase C-θ. *Eur J Biochem* **225**: 195–203

Bates SE, Currier SJ, Alvarez M and Fojo AT (1992) Modulation of P-glycoprotein phosphorylation and drug transport by sodium butyrate. *Biochemistry* **31**: 6366–6372

Bates SE, Lee JS, Dickstein B, Spolyar M and Fojo AT (1993) Differential modulation of P-glycoprotein transport by protein kinase inhibition. *Biochemistry* **32**: 9156–9164

Beck J, Handgretinger R, Klingebiel T, Dopfer R, Schaich M, Ehninger G, Niethammer D and Gekeler V (1996) Expression of PKC isozyme and MDR-associated genes in primary and relapsed state AML. *Leukemia* **10**: 426–433

Blobe GC, Sachs CW, Khan WA, Fabbro D, Stabel S, Wetsel WC, Obeid LM, Fine RL and Hannun YA (1993) Selective regulation of expression of protein kinase C (PKC) isoenzymes in multidrug-resistant MCF-7 cells. *J Biol Chem* **268**: 658–664

Budworth J, Davies R, Malkhandi J, Gant TW, Ferry DR and Gescher A (1996) Comparison of staurosporine and four analogues: their effects on growth, rhodamine 123 retention and binding to P-glycoprotein in multidrug-resistant MCF-7/Adr cells. *Br J Cancer* **73**: 1063–1068

Chambers TC, McAvoy EM, Jacobs JW and Eilon G (1990) Protein kinase C phosphorylates P-glycoprotein in multidrug resistant human KB carcinoma cells. *J Biol Chem* **265**: 7679–7686

Chambers TC, Pohl J, Raynor RL and Kuo JF (1993) Identification of specific sites in human P-glycoprotein phosphorylated by protein kinase C. *J Biol Chem* **268**: 4592–4595

Chambers TC, Pohl J, Glass DB and Kuo JF (1994) Phosphorylation by protein kinase C and cyclic AMP-dependent kinase of synthetic peptides derived from the linker region of human P-glycoprotein. *Biochem J* **299**: 309–315

Chambers TC, Germann UA, Gottesman MM, Pastan I, Kuo JF and Ambudkar SV (1995) Bacterial expression of the linker region of human *MDR1* p-glycoprotein and mutational analysis of phosphorylation sites. *Biochemistry* **34**: 14156–14162

Chaudhary PM and Roninson IB (1992) Activation of *MDR1* (P-glycoprotein) gene expression in human cells by protein kinase C agonists. *Oncol Res* **4**: 281–290

Dahllof B, Martinsson T and Levan G (1984). Resistance to actinomycin D and to vincristine induced in a SEWA mouse tumor cell line with concomitant appearance of double minutes and a low molecular weight protein. *Exp Cell Res* **152**: 415–426

Davies R, Budworth J, Riley J, Snowden R, Gescher A and Gant TW (1996) Regulation of P-glycoprotein 1 and 2 gene expression and protein activity in two MCF-7/Dox cell line subclones. *Br J Cancer* **73**: 307–315

Dong Z, Ward N, Fan D, Gupta KP and O'Brian CA (1991) In vitro model for intrinsic drug resistance: effects of PKC activators on the chemosensitivity of cultured human colon cancer cells. *Mol Pharmacol* **39**: 563–569

Fairchild CR, Kao-Shan C-S, Wang-Peng J, Rosen N, Israel MA, Malera PW, Cowan KH and Goldsmith ME (1987) Isolation of amplified and overexpressed DNA sequences from adriamycin-resistant human breast cancer cells. *Cancer Res* **47**: 5141–5148

Fine RL, Patel J and Chabner BA (1988) Phorbol esters induce multidrug resistance in human breast cancer cells. *Proc Natl Acad Sci USA* **85**: 582–586

Finkenzeller G, Marme D and Hug H (1990) Sequence of human protein kinase C alpha. *Nucleic Acids Res* **18**: 2183

Fojo A, Lebo R, Shimizu N, Chin JE, Roninson IB, Merlino GT, Gottesman MM and Pastan I (1986) Localization of multidrug resistance-associated DNA-sequences to human chromosome-7. *Somat Cell Mol Genet* **12**: 415–420

Gekeler V, Boer R, Überall F, Ise W, Schubert C, Utz I, Hofmann H, Sanders KH, SchÄchtele C, Klemm K and Grunicke H (1996) Effects of the selective bisindolylmaleimide protein kinase C inhibitor GF 109203X on P-glycoprotein-mediated multidrug resistance. *Br J Cancer* **74**: 897–905

Germann UA, Chambers TC, Ambudkar SV, Licht T, Cardarelli CO, Pastan I and Gottesman MM (1996) Characterization of phosphorylation-defective mutants of human P-glycoprotein expressed in mammalian cells. *J Biol Chem* **271**: 1708–1716

Goodfellow HR, Sardini A, Ruetz S, Callaghan R, Gros P, Mcnaughton PA and Higgins CF (1996) Protein kinase C-mediated phosphorylation does not regulate drug transport by the human multidrug resistance P-glycoprotein. *J Biol Chem* **271**: 13668–13674

Gottesman MM and Pastan I (1993) Biochemistry of multidrug resistance mediated by the multidrug transporter. *Annu Rev Biochem* **62**: 385–427

Gupta KP, Ward NE, Gravitt KR, Bergman PJ and O'Brian CA (1996) Partial reversal of multidrug resistance in human breast cancer cells by an *N*-myristoylated protein kinase C-α pseudosubstrate peptide. *J Biol Chem* **271**: 2102–2111

Hamada H, Hagiwara K, Nakajima T and Tsuruo T (1987) Phosphorylation of the $M_r$ 170,000 to 180,000 glycoprotein specific to multidrug-resistant tumor cells: effects of verapamil, trifluoperazine and phorbol esters. *Cancer Res* **47**: 2860–2865

Madden MJ, Morrow CS, Nakagawa M, Goldsmith ME, Fairchild CR and Cowan KH (1993) Identification of 5′ and 3′ sequences involved in the regulation of transcription of the human *mdr*1 gene *in vivo*. *J Biol Chem* **268**: 8290–8297

Meyers MB, Spengler BA, Chang T, Melera PW and Biedler JL (1985) Gene amplification-associated cytogenetic aberrations and protein changes in vincristine-resistant Chinese hamster, mouse and human cells. *J Cell Biol* **100**: 588–597

 © Cancer Research Campaign 1997

Morrow CS, Chiu J and Cowan KH (1992) Posttranscriptional control of glutathione S-transferase π gene expression in human breast cancer cells. *J Biol Chem* **267**: 10544–10550

O'Brian CA, Fan D, Ward NE, Seid C and Fidler IJ (1989) Level of protein kinase C activity correlates directly with resistance to adriamycin in murine fibrosarcoma cells. *FEBS Lett* **246**: 78–82

Posada J, Vichi P and Tritton TR (1989) Protein kinase C in adriamycin action and resistance in mouse sarcoma 180 cells. *Cancer Res* **49**: 6634–6639

Riordan JR, Deuchars K, Kartner N, Alon N, Trent J and Ling V (1985) Amplification of P-glycoprotein genes in multidrug resistant mammalian cell lines. *Nature* **316**: 817–819

Sachs CW, Safa AR, Harrison SD and Fine RL (1995) Partial inhibition of multidrug resistance by safingol is independent of modulation of P-glycoprotein substrate activities and correlated with inhibition of protein kinase C. *J Biol Chem* **270**: 26639–26648

Sato W, Yusa K, Naito M and Tsuruo T (1990) Staurosporine, a potent inhibitor of C-kinase, enhances drug accumulation in multidrug-resistant cells. *Biochem Biophys Res Commun* **173**: 1252–12570

Scala S, Dickstein B, Regis J, Szallasi Z, Blumberg PM and Bates SE (1995) Bryostatin 1 affects P-glycoprotein phosphorylation but not function in multidrug-resistant human breast cancer cells. *Clin Cancer Res* **1**: 1581–1587

Smith CD and Zilfou JT (1995) Circumvention of P-glycoprotein-mediated multiple drug resistance by phosphorylation modulators is independent of protein kinases. *J Biol Chem* **270**: 28145–28152

Stanwell C, Gescher A, Bradshaw TD and Pettit GR (1994) The role of protein kinase C isoenzymes in the growth inhibition caused by bryostatin 1 in human A549 lung and MCF-7 breast carcinoma cells. *Int J Cancer* **56**: 585–592

Ueda K, Cardarelli C, Gottesman MM and Pastan I (1987) Expression of a full length cDNA for the human 'MDR1' gene confers resistance to colchicine, doxorubicin and vinblastine. *Proc Natl Acad Sci USA* **84**: 3004–3008

Utz I, Hofer S, Regenass U, Hilbe W, Thaler J, Grunicke H and Hofmann J (1994) The protein kinase C inhibitor CGP 41251, a staurosporine derivative with antitumor activity, reverses multidrug resistance. *Int J Cancer* **57**: 104–110

Yu G, Ahmad S, Aquino A, Fairchild CR, Trepel JB, Ohno S, Suzuki K, Tsuruo T, Cowan KH and Glazer RI (1991) Transfection with protein kinase C-α confers increased multidrug resistance to MCF-7 cells expressing P-glycoprotein. *Cancer Commun* **3**: 181–189

Zhang F, Riley J and Gant TW (1996) Use of internally controlled reverse transcripase–polymerase chain reaction for absolute quantitation of individual multidrug resistant gene transcripts in tissues samples. *Electrophoresis* **17**: 255–260

© Cancer Research Campaign 1997